# DNA Repair Pathway Variants Are Enriched in Individuals with Biallelic AAGGG CANVAS and *RFC1* ‐Related Disease

**DOI:** 10.1002/mds.70428

**Published:** 2026-07-19

**Authors:** Xuemin Wang, Liam G. Fearnley, Kayli C. Davies, Penny Snell, Stuart Lee, Victoria E. Jackson, Justin Read, Michael Milton, Ian H. Harding, Martin B. Delatycki, David J. Szmulewicz, Paul J. Lockhart, Melanie Bahlo, Haloom Rafehi

**Affiliations:** ^1^ Walter and Eliza Hall Institute of Medical Research Melbourne Victoria Australia; ^2^ Department of Medical Biology University of Melbourne Parkville Victoria Australia; ^3^ Bruce Lefroy Centre Murdoch Children's Research Institute Parkville Victoria Australia; ^4^ Department of Paediatrics University of Melbourne Parkville Victoria Australia; ^5^ QIMR Berghofer Medical Research Institute Herston Queensland Australia; ^6^ Department of Neuroscience, School of Translational Medicine Monash University Melbourne Victoria Australia; ^7^ Victorian Clinical Genetics Services, Murdoch Children's Research Institute Royal Children's Hospital Parkville Victoria Australia; ^8^ Balance Disorders and Ataxia Service Royal Victorian Eye and Ear Hospital East Melbourne Victoria Australia; ^9^ NeuroMovement Research Program Bionics Institute Fitzroy Victoria Australia

**Keywords:** cerebellar ataxia, DNA mismatch repair, genetics, modifier, repeat expansions

## Abstract

**Background:**

Cerebellar ataxia, neuropathy and vestibular are flexia syndrome (CANVAS) and RFC1‐related disease are most commonly caused by biallelic AAGGG repeat expansions in RFC1. The high population frequency of this expansion compared to the frequency of CANVAS suggests incomplete penetrance.

**Objective:**

To determine whether polygenic risk factors influence incomplete penetrance in RFC1‐related disease.

**Methods:**

Biallelic RFC1 AAGGG status was profiled in Genomics England (GEL) and the UK Biobank (UKB) discovery cohorts and a replication Australian ataxia cohort (AAC). Polygenic score (PGS) analysis was performed with variants identified in a Huntington's Disease age at onset study.

**Results:**

We identified 56 (GEL) and 18 (UKB) biallelic individuals with ataxia and/or neuropathy, and 26 (GEL) and 207 (UKB) age‐matched biallelic carriers with no report of ataxia or neuropathy. Meta‐analysis of GEL and UKB identified elevated PGS in biallelic affected individuals (OR = 1.43, *p* = 2.2 × 10^−3^) compared to controls, but not in the biallelic carriers (OR = 1.01, *P* = 0.92). The variant rs245100, upstream MSH3, was the biggest contributor to this elevated risk (OR = 2.35, adjusted *p* = 0.02). Replication in the AAC showed elevated PGS in individuals with molecular confirmation of CANVAS or RFC1‐related disease (*n* = 54) (OR = 1.93, *P* = 4.4 × 10^−5^), and enrichment of rs245100 (OR = 4.24, *p* = 1.0 × 10^−4^).

**Conclusion:**

These results indicate that modifier variant burden alters disease penetrance and presentation in RFC1‐related disease and determines an increased incidence of ataxia and neuropathy in biallelic individuals. © 2026 The Author(s). *Movement Disorders* published by Wiley Periodicals LLC on behalf of International Parkinson and Movement Disorder Society.

Cerebellar ataxia, neuropathy, and vestibular areflexia syndrome (CANVAS) is an adult‐onset neurological disorder presenting with cerebellar ataxia, neuropathy, and vestibular hypofunction. For most affected individuals a chronic cough generally precedes the onset of ataxia by decades.^1^, ^2^ CANVAS is one of the most common forms of inherited late‐onset ataxia worldwide, affecting ~1 in 20,000 individuals. It is caused primarily by biallelic AAGGG repeat expansions (RE) in *RFC1*,^3^, ^4^ as well as other pathogenic motifs that are less common, such as the ACAGG motif described in Asia‐Pacific populations.^5^, ^6^

More recently, the term “*RFC1*‐related disorder” has been used in recognition that biallelic expansion can cause additional clinical phenotypes, including isolated ataxia, neuropathy, and cough, in addition to symptoms such as autonomic dysfunction and parkinsonism.^7^ The polymerase chain reaction (PCR)–validated carrier frequency of the *RFC1* AAGGG RE may be as high as 1 in 16, as determined in a healthy Australian cohort (n = 269),^8^ consistent with other studies.^9^ The range for the expected number of homozygous individuals is between 1 in 1111 and 1 in 1024 in PCR‐validated cohorts, and between 1 in 2500 and 1 in 716 in biobank‐based studies,^6^, ^10^ all of which are higher than would be expected under a full penetrant model of CANVAS. In addition, incomplete penetrance may affect disease manifestation. Hardy–Weinberg equilibrium holds for the *RFC1* AAGGG allele and genotype frequencies,^11^ suggesting that no overt selection against the AAGGG allele exists. This suggests underdiagnosis in the general population and/or incomplete penetrance affects disease manifestation.

In recent years, multiple genome‐wide association studies (GWAS) have identified modifier variants that play a key role in the modulation of age at onset and disease severity of otherwise monogenic repeat expansion disorders (RED) with CAG/CTG and AGAGGG motifs such as Huntington's disease (HD), X‐linked dystonia parkinsonism, some spinocerebellar ataxias, and myotonic dystrophy 1.^12^, ^13^, ^14^, ^15^, ^16^, ^17^ Analysis of closest neighbor genes demonstrates an overrepresentation of DNA mismatch repair (MMR) pathway genes, including *MSH3*, which is thought to modulate age at onset and disease severity by driving increased rates of somatic expansions, best characterized in HD.^18^

In *RFC1*‐related disease, evidence suggests that earlier symptom onset and greater disease severity are associated with larger repeat size of both alleles.^19^ To the best of our knowledge, no studies have investigated genetic modifiers in *RFC1*‐related disorder, CANVAS, or any other disorders caused by pentamer RE motifs. It is currently difficult to perform a GWAS of age at onset in individuals with CANVAS due to the rarity of the disease, resulting in small sample sizes in targeted cohorts. Attempts to increase cohort numbers by leveraging large genomic biobanks present additional issues, including the absence of long‐read or PCR‐verified biallelic *RFC1* AAGGG status and allele size, and incomplete or missing age‐at‐onset data. However, short‐read sequencing has been proven to be highly sensitive and specific for the detection of *RFC1* REs, even if not fully capable of dissecting the size of the repeat.^8^, ^20^, ^21^

We hypothesize that somatic mutation burden, driven by variants in MMR genes, is predictive of incomplete penetrance in *RFC1*‐related disease. Therefore, in this study, we generated polygenic scores (PGS) from previous modifier GWAS from HD and assessed their relevance in three groups of individuals in two large biobanks and an Australian clinical ataxia cohort: (1) affected individuals who are biallelic for *RFC1* AAGGG REs, (2) biallelic *RFC1* AAGGG RE carriers who are not reported to have ataxia or neuropathy, and (3) controls who are not biallelic for *RFC1* AAGGG.

## Patients and Methods

### Ethics and Consent for Studies

The Royal Children's Hospital Human Research Ethics Committee (HREC 28097) and the Walter and Eliza Hall Institute of Medical Research (HREC 18/06 and 24/10) approved the study.

### Study Cohorts

A diagram outlining the study design and cohorts is shown in Figure 1. The discovery cohorts included Genomics England^22^ (GEL) 100,000 Genomes Project version 19 and UK Biobank (UKB). Full details of the cohort selection are presented in the Supplementary Methods and Table S1.

**FIG. 1:**
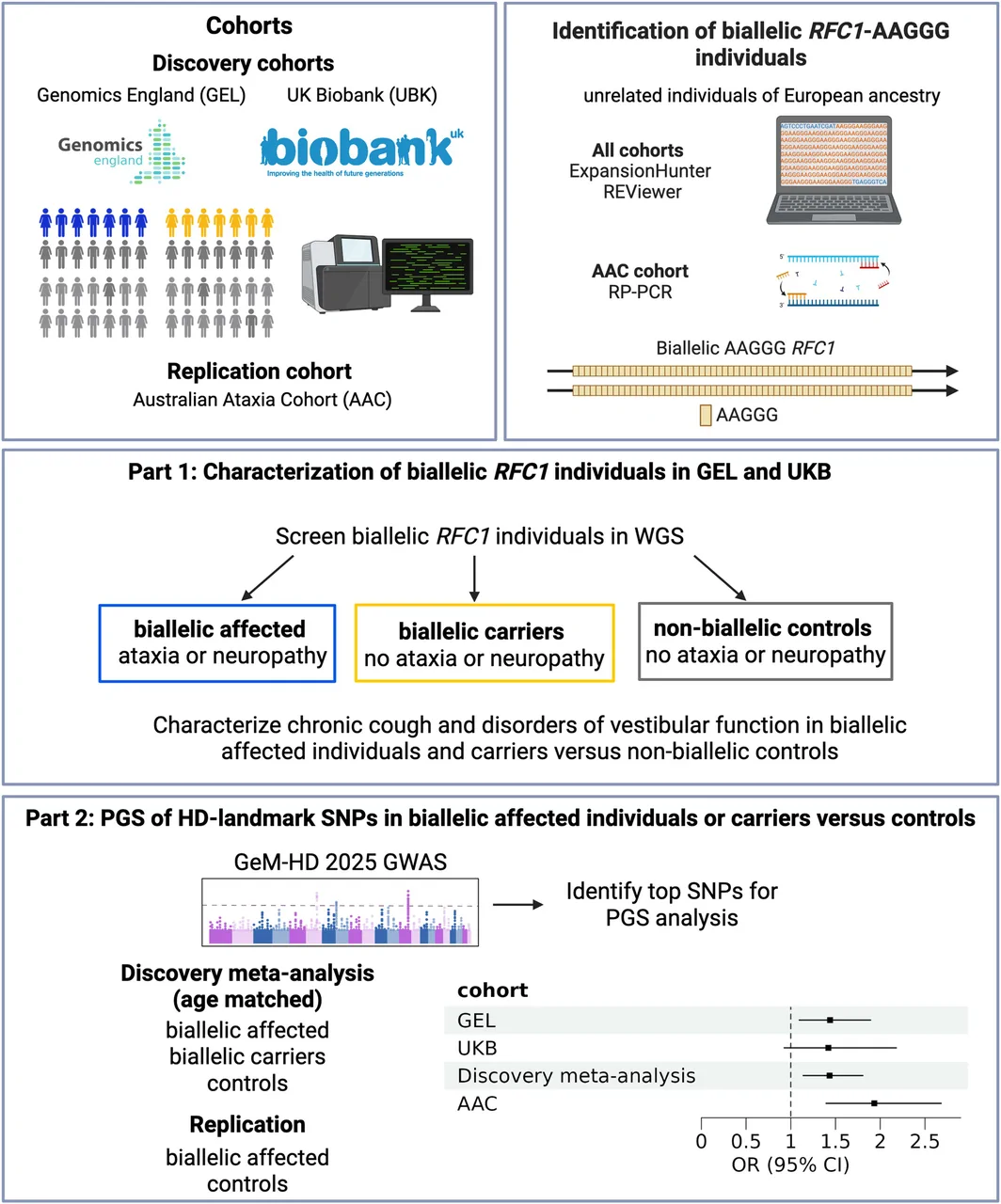
Study design. [Color figure can be viewed at wileyonlinelibrary.com]

Briefly, the study included age‐matched, unrelated participants of European ancestry from GEL and UKB with or without symptoms of ataxia or neuropathy. Two separate control cohorts were prepared for the symptom and the PGS‐focused analyses. Participants with neurological disorders other than ataxia or neuropathy were included in the symptomatic analysis but excluded from the PGS analysis to minimize bias associated with undetected pathogenic RED.

The Australian ataxia cohort (AAC) was used for replication, which included individuals with a clinical diagnosis of adult‐onset cerebellar ataxia or neuropathy and Long range (LR)‐PCR‐validated biallelic status. A cohort of healthy adults recruited from multiple sites across Australia served as controls. All individuals underwent short‐read Illumina whole genome sequencing (WGS), as previously described.^3^, ^8^, ^20^ Biallelic status at the *RFC1* AAGGG locus was comprehensively profiled in both affected and unaffected individuals as previously described using two orthogonal approaches.^8^ None of the controls were biallelic at the *RFC1* AAGGG locus. Genetic ancestry of AAC participants and controls was determined using *Peddy*.^23^

### Determination of Biallelic *RFC1* AAGGG Repeat Expansion Status

*RFC1* AAGGG repeat size was genotyped using *ExpansionHunter*, version *5.0.0*,^24^ for each participant in GEL and UKB identified earlier and confirmed using *REViewer*.^25^ A subset of individuals in GEL was validated using long‐read sequencing data. Full details are provided in Supplementary Methods.

### Symptom Analyses

We compared the incidence of cough and vestibular dysfunction in biallelic individuals and carriers compared to controls in GEL and UKB (full details in Supplementary Methods). Association testing was carried out using generalized linear models implemented in the base R package *stats*, version *4.5.1*.

### Polygenic Score Analyses

PGS analysis, single‐nucleotide polymorphism (SNP) associations, and age‐at‐onset analysis were performed in the GEL and UKB cohorts, and replicated in the AAC cohort (full details in Supplementary Methods). Briefly, a final set of nine independent lead/proxy variants from the latest Genetic Modifiers of Huntington's Disease Consortium study^12^ was included to generate PGSs (Table S3). Variant weights were inverted from the β values, such that positive weights corresponded to more severe or faster progressing HD. Associations of disease risk between normalized PGS of biallelic affected individuals and controls, and biallelic unaffected carriers and controls, were tested using Firth's logistic regression,^26^ with adjustment for the first six ancestry principal components (PC). Meta‐analysis was performed using a fixed‐effect model.

## Results

### Cohort Description

The study overview is summarized in Figure 1. We identified two large‐scale genomics cohorts for the discovery arm of this study: GEL and UKB. These two cohorts have very different recruitment strategies. The GEL 100,000 Genomes Project rare diseases program recruited participants with rare diseases of unknown genetic origin and included a subprogram focused on neurological conditions (N = 16,005). In contrast, the UKB is a population‐based cohort, which has been shown to be healthier than expected.^27^ There were more participants with ataxia and/or neuropathy in GEL than in UKB (Fig. S1A,B). For genetic analysis, we identified 56 unrelated individuals with ataxia and/or neuropathy with European ancestry with biallelic pathogenic *RFC1* AAGGG REs (biallelic affected individuals) and 26 age‐matched unrelated European participants who are biallelic for *RFC1* AAGGG but do not have ataxia or neuropathy (biallelic carriers) in GEL. In UKB, we identified 18 biallelic affected individuals and 207 age‐matched biallelic carriers (Table 1). For the symptom‐based analysis, we identified 3248 (GEL) and 115,177 (UKB) age‐matched unrelated controls of European ancestry who have no or one copy of the *RFC1* AAGGG pathogenic allele and do not have ataxia or neuropathy. Overall, biallelic participants, regardless of disease status, account for 2.5% of participants in GEL and 0.2% in UKB, with the prevalence of biallelic individuals with ataxia and/or neuropathy being 1.7% in GEL and 0.02% in UKB. Further exclusion of participants with neurological diseases led to 1854 controls in GEL and 82,403 controls in the UKB for PGS analysis.

**TABLE 1:** Sample sizes in the discovery and replication cohorts

Cohort	Biallelic affected individuals	Biallelic carriers*	Controls for symptom analysis**	Controls for PGS analysis***
GEL	56	26	3248	1854
UKB	18	207	115,177	82,403
AAC	54			229

Abbreviations: PGS, polygenic score; GEL, Genomics England; UKB, UK Biobank; AAC, Australian ataxia cohort.

* Biallelic carriers were age matched to biallelic affected individuals.

** Participants with any diseases of the nervous system were included in controls for symptom analysis but excluded from controls in PGS analysis in both GEL and UKB.

*** Controls were age matched in GEL and UKB but not able to be age matched in AAC.

Biallelic *RFC1* individuals account for 10.5% of individuals in GEL with both ataxia and neuropathy; however, no participants in the UKB had both ataxia and neuropathy, regardless of *RFC1* status (Fig. S1A–D). Only 1.6% (GEL) and 3.3% (UKB) of individuals with ataxia without neuropathy were biallelic for *RFC1* (Fig. S1C,D), whereas 1.0% (GEL) and 1.9% (UKB) of individuals with neuropathy but without ataxia were biallelic (Fig. S1A–D).

In the UKB, the mean age of the biallelic affected group is 75.1 years, with a mean age at ataxia or neuropathy diagnosis of 67.1 years and age at last clinical investigation of 73.1 years (Fig. S2A). We show that, after age matching, the age at last investigation in the controls and biallelic carriers is older than that at ataxia or neuropathy diagnosis of the biallelic affected individuals (Fig. S2D). In GEL, the age distribution of the biallelic carriers reflected the broader age distribution and was skewed toward a younger age demographic. The biallelic carriers were only partially age matched, and the group is younger (mean age 61.6 years, *P* = 0.02) than the age at ataxia or neuropathy diagnosis of biallelic affected individuals (mean age 66.8 years). Therefore, in GEL, but not in the UKB, the biallelic carrier group may be enriched for individuals who could develop ataxia and/or neuropathy as they age.

Despite recent advances, the *RFC1* RE remains difficult to accurately characterize in short‐read sequencing. Particularly, the sequencing quality at this site is often poor due to the high G‐base content, the motif composition is highly variable, and the pathogenic allele length is too long to be accurately sized.^5^, ^6^, ^8^, ^20^, ^28^, ^29^, ^30^ We therefore utilized *REViewer* to visually inspect all candidate individuals genotyped as biallelic AAGGG by *ExpansionHunter* to confirm the presence of the motif on both alleles. We show that the true positive rate was higher in individuals with longer *ExpansionHunter* allele genotype estimates (Table S2). Specifically, a true positive rate of 42.8% (UKB) and 62.7% (GEL) was observed in individuals with a shorter allele genotyped as ≥55 repeats, compared to 4.4% (UKB) and 1.2% (GEL) in individuals with a shorter allele genotyped as ≥30 but <40 repeats, indicating that a manual review of *RFC1* AAGGG allele calls from *ExpansionHunter* is essential.

We then leveraged long‐read sequencing data from GEL to further profile the accuracy of our biallelic status calls. Using matched long‐read and short‐read sequencing data available in GEL for six participants, we confirmed that biallelic and monoallelic *RFC1* AAGGG status determined using *ExpansionHunter* genotyping followed by visual inspection of *REViewer* plots were all consistent with the long‐read WGS data (Fig. S3).

### Higher Risk of Cough and Disorders of Vestibular Function in Biallelic Individuals

We hypothesized that there is a previously unrecognized burden of *RFC1*‐related disease among biallelic carriers. To investigate this, we examined two common but nonspecific clinical features of CANVAS: cough (R05) and vestibular dysfunction (H81).

As expected, we observed increased incidence of cough in biallelic affected individuals compared to controls in both GEL (odds ratio [OR] = 4.62, *P* = 1.0 × 10^−4^) and UKB (OR = 13.27, *P* = 5.2 × 10^−6^) (Fig. 2A,B). However, we also observed a higher incidence of cough in biallelic carriers compared to controls in UKB (OR = 5.80, *P* = 2.4 × 10^−15^) and in GEL (OR = 2.31, *P* = 0.26), with a reduced OR. However, the latter result was not significant, likely due to smaller sample sizes in GEL (n = 26). Meta‐analysis of the discovery cohorts using the fixed‐effect model showed biallelic affected individuals had an increased incidence of cough of OR = 6.51 (*P* = 6.9 × 10^−9^) and biallelic carriers an increased risk of OR = 5.38 (2.6 × 10^−15^) compared to age‐matched controls.

**FIG. 2:**
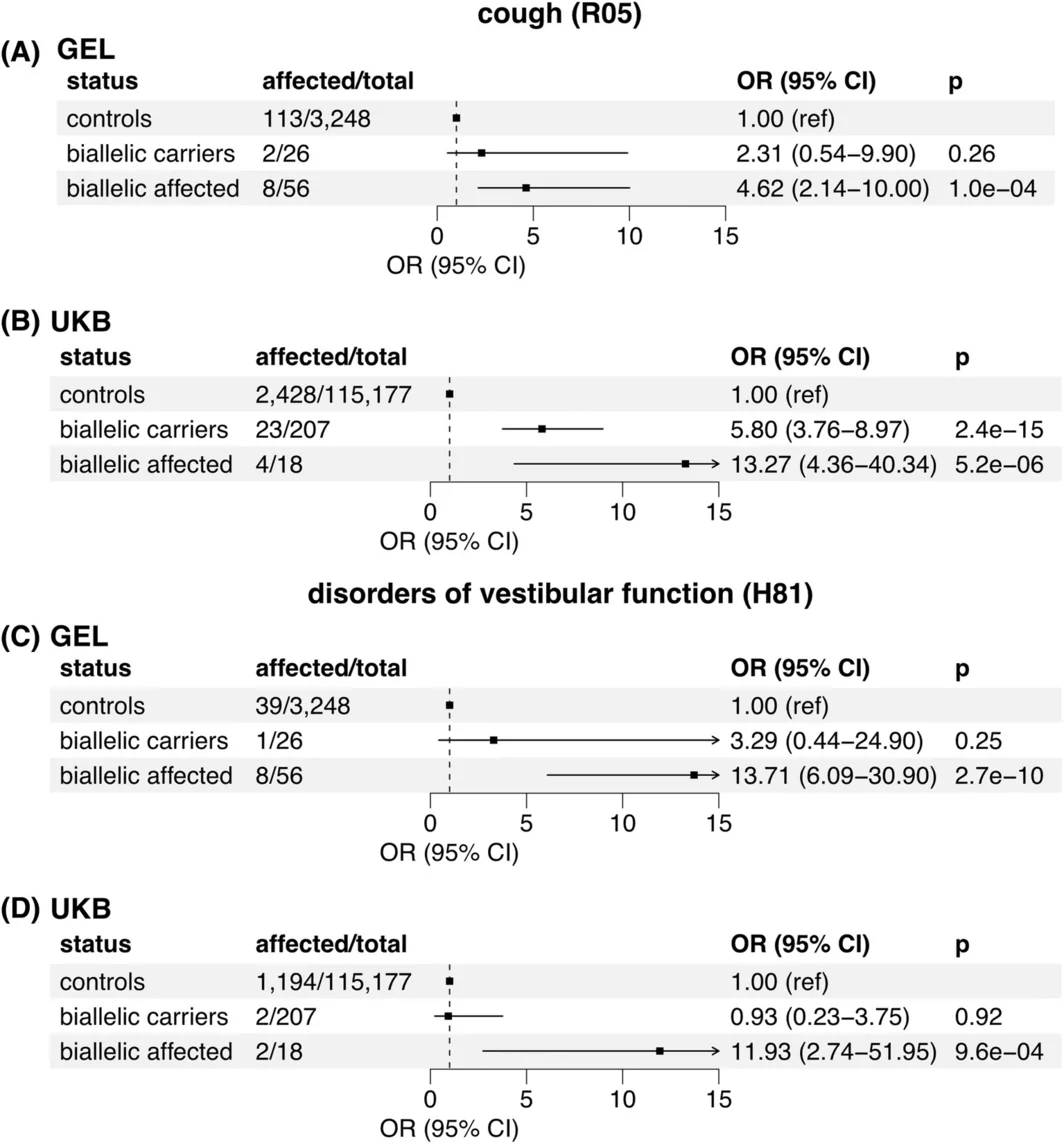
Risk of developing cough and disorders of vestibular function. The risk of developing cough and disorders of vestibular function biallelic affected individuals and carriers compared to controls in **(A, C)** Genomics England (GEL) and **(B, D)** UK Biobank (UKB), respectively. OR, odds ratio; ref indicates the controls group is used as the reference.

Risk of disorders of vestibular functions was elevated in both discovery cohorts (OR = 13.71, *P* = 2.7 × 10^−10^ in GEL and OR = 11.93, *P* = 9.6 × 10^−4^ in UKB) in biallelic affected individuals compared to controls, whereas no significant increase in risk was observed in biallelic carriers (Fig. 2C,D). The number of reported bilallelic affected individuals and carriers with cough is much higher than that reported as having vestibular dysfunction, with cough likely showing ascertainment bias.

### Increased *RFC1* ‐Related Disease Risk Associated with Higher PGS

We generated PGS of age at total functional capacity score of 6 (TFC6) of HD in the discovery cohorts. A higher PGS is associated with an increased burden of DNA repair pathway SNPs that were found to reduce age at TFC6 in HD. Due to the small cohort sizes, only variants with Minor Allele Frequency (MAF) >10% were included in the analysis. Biallelic affected individuals exhibited higher mean PGS than controls in both discovery cohorts (Fig. 3A,B); the difference in mean PGS between biallelic affected individuals and controls was significant in GEL (adjusted *P* = 0.02) but not in UKB (adjusted *P* = 0.40; Fig. S3A,B). PGS of biallelic carriers was significantly higher than that of controls in GEL but not in UKB. This could be due to the overall lower mean age of biallelic carriers in GEL, who may go on to develop symptoms as they age. Furthermore, 13 of the 26 biallelic carriers were probands *recruited through rare disease programs*. However, only 3 of these individuals have disorders known to be potentially caused by REs (muscular dystrophy, intellectual disability, Parkinson's disease) (Table S4). Therefore, there is no evidence that the increased burden observed in this group is attributable to enrichment of other pathogenic REs in this group.

**FIG. 3:**
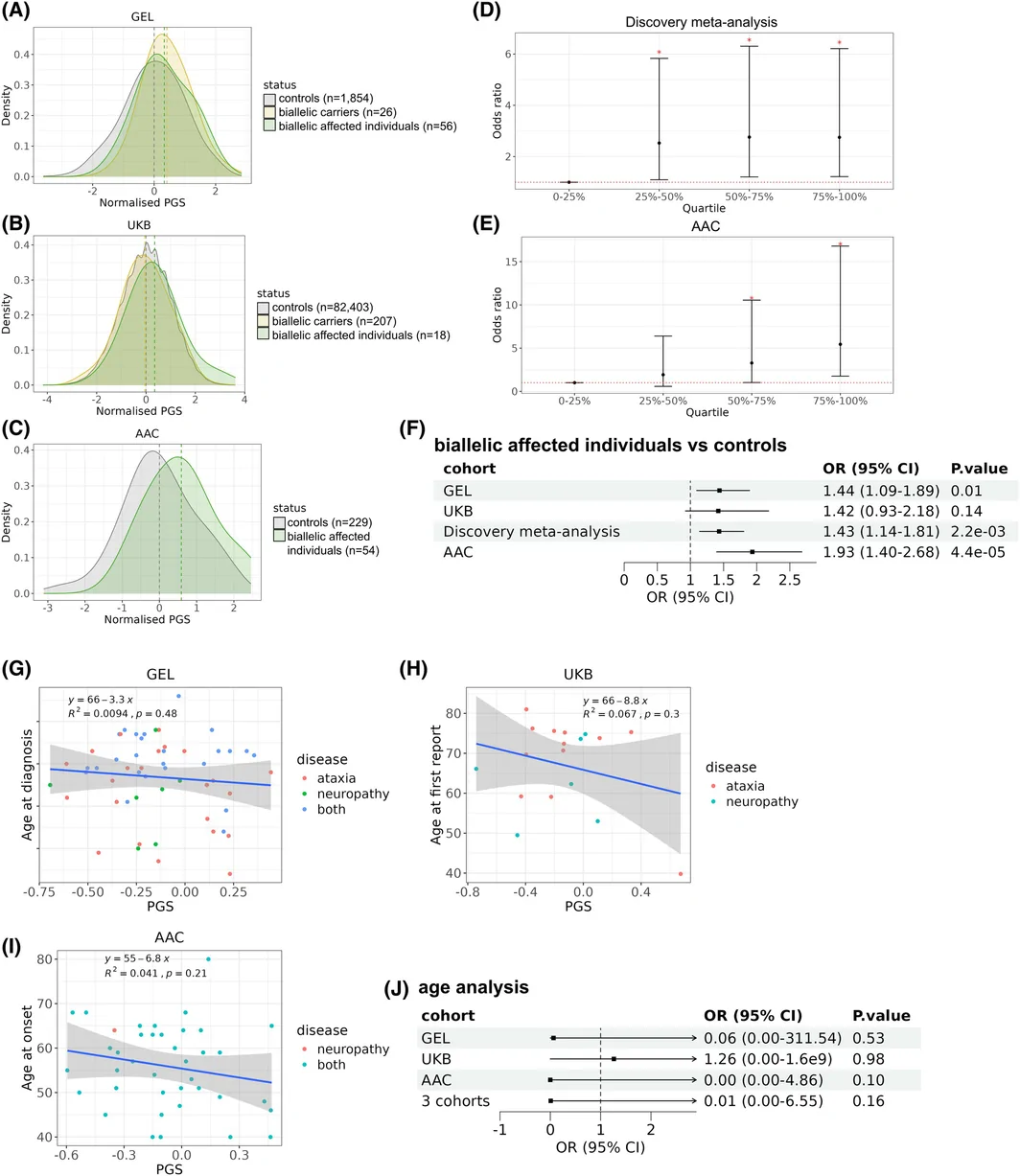
Polygenic scores (PGS) in differentiating biallelic affected individuals, biallelic carriers, and controls in the discovery and replication cohorts. Density plots of PGS of biallelic affected individuals, biallelic carriers, and controls in **(A)** Genomics England (GEL), **(B)** UK Biobank (UKB), and **(C)** Australian ataxia cohort (AAC). The AAC cohort did not include biallelic carriers. Odds ratio (OR) of *RFC1*‐related disease risk of the other three quartiles of higher PGS compared to the bottom quartile with lowest PGS in the **(D)** discovery cohort meta‐analysis and the **(E)** AAC replication cohort. **(F)** Forest plot of the risk (OR) of developing *RFC1*‐related disease with one standard deviation (SD) increase in PGS. **(G)** Age at diagnosis of the specific rare disease that the participant was recruited as a proband for was used as surrogate of age at onset of *RFC1*‐related disease in GEL; **(H)** age at first report of ataxia or neuropathy was used as surrogate of age at onset in UKB; **(I)** age at onset of symptoms other than cough was presented in AAC. The first six genetic principal components (PC) and disease type (ataxia, neuropathy, or both) were adjusted for in the robust linear models to assess the relationship between PGS and age at onset (or surrogate). **(J)** Meta‐analysis results of the three cohorts. Display of exact age at diagnosis of individuals in GEL is not allowed to protect participants; therefore, age at diagnosis in GEL was omitted from the *x*‐axis in graph (G) to comply with GEL policy.

We then split participants in GEL and UKB into the quartiles of the standardized PGS and estimated the risk of developing *RFC1*‐related disease of the top three quartiles compared to the first quartile, which has the lowest PGS. Meta‐analysis of the discovery cohorts showed that participants in all three quartiles with higher PGS exhibited increased *RFC1*‐related disease risk (OR = 2.53, *P* = 0.03 in the 25%–50% quartile; OR = 2.76, *P* = 0.02 in the 50%–75% quartile; OR = 2.75, *P* = 0.01 in the 75%–100% quartile; Fig. 3D).

We next sought to replicate these findings in the AAC. We observed a higher mean PGS in biallelic affected individuals than controls (*P* = 5.8 × 10^−5^) in the AAC (Fig. 3C). There was also a trend of increased PGS quartiles associated with higher *RFC1*‐related disease risk in the AAC; both participants in the third (50%–75%, OR = 3.28, *P* = 0.04) and last (75%–100%, OR = 5.44, *P* = 0.003) quartiles of PGS exhibited elevated risk of developing *RFC1*‐related disease compared to the first quartile (Fig. 3E).

We also implemented Firth's logistic regression to evaluate the association between *RFC1*‐related disease risk and PGS and found the relationship to be positive in all cohorts (Fig. 3F). Meta‐analysis of the discovery cohorts between biallelic affected individuals and controls showed *RFC1*‐related disease risk increased by 43% with a 1 standard deviation increase in PGS (OR = 1.43, *P* = 2.2 × 10^−3^). This signal was replicated in the AAC but with a stronger signal (OR = 1.93, *P* = 4.4 × 10^−5^). Meta‐analysis of the discovery cohorts showed that there is no association between the PGS and asymptomatic biallelic carriers compared to controls (OR = 1.01, *P* = 0.92; Fig. S4C). Furthermore, there was no association between *RFC1*‐related disease risk and changes in polygenic risk scores of asthma or Inflammatory Bowel Disease (IBD) used as negative controls (Fig. S5). We decomposed the PGS, testing the nine variants included in the PGS calculation separately using *χ* ^2^ tests for independence. Only one variant, upstream of *MSH3*, rs245100, was significantly associated with *RFC1*‐related disease risk (adjusted *P* = 0.02); biallelic affected individuals with the effect allele (G) had a 2.35 times higher risk of developing *RFC1*‐related disease compared to controls (Table 2). This finding was replicated in the AAC cohort (OR = 4.24, *P* = 1.0 × 10^−4^), continuing the pattern of stronger effect sizes observed in this cohort compared with the two discovery cohorts.

**TABLE 2:** *Enrichment of biallelic affected individuals in SNP genotypes revealed by χ* ^*2*^ *test results and risk of RFC1‐related disease compared to controls for the nine variants included in polygenic score calculation*

SNP_EA	Candidate gene	GeM‐HD 2025			Discovery cohorts			
		MAF	β^a^	*P*	*χ* ^2^	df^b^	Adjusted *P*‐value^c^	OR^d^
rs245100_G	*MSH3*	0.25	0.23	5.63E‐57	13.37	NA	0.02	2.35
rs3512_C	*FAN1*	0.31	−0.22	1.08E‐53	0.48	2	0.79	1.17
rs1650735_A	*MSH3*	0.16	−0.22	1.55E‐34	1.69	NA	0.67	0.72
rs10411910_T	*LIG1*	0.13	−0.15	7.99E‐15	0.74	NA	0.79	0.98
rs12531376_T	*PMS2*	0.16	−0.14	9.59E‐16	6.53	NA	0.16	0.46
rs2345058_C	*PMS2*	0.41	−0.12	1.82E‐19	1.29	2	0.67	0.77
rs6434365_C	*PMS1*	0.21	0.12	5.85E‐14	2.06	NA	0.63	1.17
rs12365516_T	*CCDC82*	0.2	−0.11	9.99E‐13	2.97	NA	0.49	0.83
rs165666_A	*MED15*	0.21	0.09	5.03E‐09	5.49	NA	0.19	1.39

Abbreviations: SNP, single‐nucleotide polymorphism; EA, effect allele; GeM‐HD, Genetic Modifiers of Huntington's Disease Consortium; OR, odds ratio; HD, Huntington's disease; NA, Not Applicable.

^a^ β values were inverted from the original paper, such that positive weights corresponded to decreasing functional capacity score, or more severe or faster progressing HD.

^b^ The degrees of freedom are NA when any expectation in the contingency table is <5 and the *P*‐value is computed by Monte Carlo simulation.

^c^ Benjamini–Hochberg false discovery rate.

^d^ Odds ratio of disease risk for participants who are heterozygous or homozygous for the effect allele compared to participants who are homozygous for the reference allele.

### Earlier Age at Onset/Diagnosis Suggestively Associates with Higher PGS

We next investigated the association between PGS and age at symptom onset, using age at diagnosis in GEL and age at first report in UKB as a proxy of age at disease onset. Results showed a negative trend between age at onset and PGS in all three cohorts, though the association was nonsignificant (Fig. 3G–J). Meta‐analysis of the three cohorts also showed no significant association between PGS and age at onset but retained the expected negative trend of poorer somatic mutation rate control (high PGS) and earlier age at onset (or surrogates thereof ).

## Discussion

Biallelic AAGGG REs in *RFC1* primarily cause *RFC1*‐related disease, which includes CANVAS.^11^ Age at disease onset in *RFC1*‐related disease is highly variable and not fully explained by the RE size.^19^ In this study, leveraging data in two large biobanks, we identified participants harboring biallelic *RFC1* AAGGG REs both with and without a diagnosis of ataxia and/or neuropathy. We found that *RFC1*‐related disease risk is associated with increased burden of DNA mismatch repair pathway variants (as measured by PGS) in the discovery cohorts and validated this association in a replication cohort (AAC) that encompassed individuals with clinically confirmed CANVAS or more broadly *RFC1*‐related disease. Our results indicate that MMR pathway variants, associated with increased somatic mutation burden in other REDs, may play a role in the development of *RFC1*‐related disease, especially CANVAS. This is the first demonstration of the role of previously discovered common genetic variants that affect somatic mutation rate in CAG RED also playing a role in a recessively inherited pentamer RED. This indicates that a GWAS with sufficient power should replicate at the very least the signal at rs245100, upstream of *MSH3*. This variant is widely accepted in the literature to be tagging *MSH3*.^12^, ^17^ Although not achieving statistical significance, other signals with large β values may replicate in larger cohorts, such as the protective variant rs12531376 in *PMS2*, which is depleted in our discovery cohort. Other SNPs showed no signal, despite having large effect sizes in the GeM‐HD GWAS, suggesting that some MMR pathway variants may be disease or motif specific.

The frequency of individuals with biallelic *RFC1* AAGGG alleles is greater than the prevalence of individuals with CANVAS. In GEL, we observe a high proportion of biallelic individuals (2.5%), which is attributed to the targeted recruitment of individuals with ataxia and neuropathy phenotypes. The majority of biallelic individuals in GEL have cerebellar ataxia and/or neuropathy, suggesting a CANVAS diagnosis. In contrast, the UKB is a much larger population cohort, with a bias toward recruiting healthier/more able‐bodied individuals. In the UKB, only 0.2% of individuals are biallelic, and 0.02% are biallelic and have features of ataxia or neuropathy, again supporting a clinical suspicion of CANVAS or *RFC1*‐related disease. The frequency of biallelic *RFC1* AAGGG REs estimated in this study was consistent with previous reports.^6^, ^10^, ^11^

One limitation of our study is the reliance on short‐read data to determine biallelic AAGGG status in the discovery cohorts. It is well known that the *RFC1* locus is complex and is susceptible to false‐positive calls. Our findings indicate estimates utilizing *ExpansionHunter* have a false‐positive rate of about 50%, even when using a stringent genotype cutoff of ≥55 repeats for both alleles. To mitigate this risk, we manually inspected the locus using *REViewer* for all potential biallelic individuals identified using *ExpansionHunter* to confirm the AAGGG allele motif composition, which we validated with long‐read sequencing for a subset of individuals in GEL. We also do not attempt to estimate the allele size, as the AAGGG RE is too large to be accurately sized in short‐read data.^3^, ^6^, ^8^ Using this approach, our results are concordant with a recent study that also profiled AAGGG status in probands in GEL, which were then confirmed using PCR for a subset of individuals, showing a very low false‐positive rate.^11^ Finally, we use a validated CANVAS and *RFC1*‐related disease cohort as the replication cohort, in which we previously confirmed biallelic AAGGG status using repeat‐primed and long‐range PCR.

We identified biallelic *RFC1* AAGGG carriers who did not have ataxia or neuropathy in both the discovery cohorts. These individuals, who were age matched to the biallelic affected individuals, exhibited enrichment for *RFC1*‐related disease phenotypes that are often associated with the early disease stage of *RFC1*‐related disease, such as chronic cough, indicating either (1) incomplete penetrance, (2) potential underdiagnosis of *RFC1*‐related disease, or (3) a potential for delayed disease onset in this group.

Our understanding of the phenotypic spectrum of *RFC1*‐related disease is continually expanding. Apart from the classical triad of CANVAS symptoms, other clinical features have also been reported. In this study, we found both biallelic *RFC1‐*affected individuals and carriers had a higher risk of cough than controls in the discovery cohorts. This suggests that some biallelic carriers may have undiagnosed *RFC1*‐related disease, with chronic cough as the only symptom to date, and may or may not progress to develop *RFC1*‐related disease or CANVAS later in life. We note that cough is a common symptom, and not all incidences of cough in biallelic individuals will be attributed to *RFC1*.

In summary, we identified both symptomatic biallelic *RFC1* AAGGG individuals and biallelic carriers who did not have cerebellar ataxia or neuropathy in two large biobanks (UKB and GEL) and an Australian clinical ataxia cohort. We show that affected individuals with biallelic *RFC1* RE exhibited higher PGS compared to controls, but unaffected biallelic carriers do not. Our results suggest propensity to somatic mutations related to DNA mismatch repairs (as measured by PGS) may explain incomplete penetrance of *RFC1* AAGGG REs. This study is the first to demonstrate that previously discovered disease‐modifying common genetic variants in CAG RED also play a role in an AAGGG expansion‐related disorder. This work also provides a strong rationale to conduct a GWAS on age at diagnosis and various symptoms of *RFC1*‐related disease such as CANVAS.

## Author Roles

(1) Research Project: A. Conception, B. Organization, C. Execution; (2) Statistical Analysis: A. Design, B. Execution, C. Review and Critique; (3) Manuscript Preparation: A. Writing of the First Draft, B. Review and Critique.

X.W.: 1A, 1B, 1C, 2A, 2B, 2C, 3A, 3B.

L.G.F.: 2B, 2C, 3B.

K.C.D.: 2B, 2C, 3B.

P.S.: 2B, 2C, 3B.

S.L.: 2B, 2C, 3B.

V.E.J.:2B, 2C, 3B.

J.R.: 1B, 2C, 3B.

M.M.:2B, 2C, 3B.

I.H.H.: 1B, 2C, 3B.

M.B.D.: 1B, 2C, 3B.

D.J.S.: 1B, 2C, 3B.

P.J.L.: 1B, 2C, 3B.

M.B.: 1A, 1B, 2A, 2C, 3B.

H.R.: 1A, 1B, 1C, 2A, 2B, 2C, 3A, 3B

## Financial Disclosures and Conflicts of Interest

Author disclosures are available in the Supporting Information.

## Supporting information


**Figure S1.** Number of biallelic affected individuals identified by disease types in three cohorts. Total number of individuals with ataxia and/or neuropathy in **(A)** Genomics England (GEL) and **(B)** UK Biobank (UKB); number of unrelated biallelic affected individuals with European ancestry in **(C)** GEL, **(D)** UKB, and **(E)** Australian ataxia cohort (AAC), with ataxia, neuropathy, or both. Values in parentheses are the percentage of unrelated biallelic affected individuals in the total number of individuals by disease; for example, 25 biallelic affected individuals with neuropathy and ataxia account for 10.5% of 238 individuals with both diseases in GEL.
**Figure S2.** Age matching in UK Biobank (UKB) and Genomics England (GEL) in the final cohort for the PGS analysis. Comparison of the current age, age at last investigation, and age at ataxia or neuropathy last diagnosis (where relevant) in the UKB is shown for **(A)** biallelic affected individuals, **(B)** biallelic carriers, and **(C)** controls. **(D)** Plot shows the comparison of the age at last investigation in UKB of biallelic carriers and controls compared to the age at ataxia or neuropathy diagnosis. In GEL, the box plot shows the comparison of current age in biallelic affected individuals, biallelic carriers, and controls, as well as the age at ataxia or neuropathy diagnosis for the biallelic affected individuals. All current ages are calculated for the year 2025. *P*‐values are from unpaired Wilcoxon tests. Outliers not plotted.
**Figure S3.** Comparison of the detection of *RFC1*:AAGGG alleles in short‐ and long‐read whole genome sequencing (WGS) data in individuals from Genomics England (GEL). **(A)** Plots show the allele size estimates for *RFC1*:AAGGG alleles detected in ONT (top) and PacBio (bottom) WGS sequencing data. Blue symbols indicate individuals called as biallelic by EH5 and *REViewer* in the short‐read data, whereas red indicates individuals with a single allele. Triangles indicate individuals identified as biallelic in ONT or PacBio, whereas circles indicate those that have a single AAGGG allele. The data are divided by allele status in affected individuals and controls as determined by short‐read sequencing, and other indicates individuals who are in the rare disease group in GEL but were not included in the final cohort in this study (due to factors such as relatedness, ethnicity, and age as detailed in the “Methods” section). **(B)** Plots show a comparison of allele size for individuals sequenced on both the ONT and PacBio platforms, with red indicating the *RFC1* AAGGG allele status determined by EH5 and *REViewer*.
**Figure S4. (A, B)** Differences in the mean polygenic score (PGS) among biallelic affected individuals, carriers, and controls in the discovery cohorts and **(C)** *RFC1‐*related disease risk of biallelic carriers compared to controls estimated using Firth's logistic regression.
**Figure S5.** Forest plot of odds ratio estimated by Firth's logistic regression using PGS of IBD/asthma in the discovery and validation cohort in biallelic affected individuals (top) and carriers (bottom).

**Data S1:** COI_disclosure.

## Data Availability

Data from the National Genomic Research Library (NGRL) used in this research are available within the secure Genomics England Research Environment. Access to NGRL data is restricted to adhere to consent requirements and protect participant privacy. Data used in this research include Genomics England rare diseases program data, accessed under project RR1261. Access to NGRL data is provided to approved researchers who are members of the Genomics England Research Network, subject to institutional access agreements and research project approval under participant‐led governance. For more information on data access, visit https://www.genomicsengland.co.uk/research. Data from the UKB can be accessed through an application process, via https://ukbiobank.ac.uk. The AAC cohort sequencing data cannot be shared due to ethical/privacy issues, but specific requests for information will be considered by the authors. The code for analyses in this study is publicly available at https://github.com/bahlolab/RFC1-related-disease-pgs.
