# Supplementary material for: DNA Repair Pathway Variants Are Enriched in Individuals with Biallelic AAGGG CANVAS and RFC1 ‐Related Disease
Source: Mov Disord. 2026 Jul 19;41(10):2745–54. doi: 10.1002/mds.70428 (PMC13686117; doi:10.1002/mds.70428)
Supplement: Supplementary file 2 [file MDS-41-2745-s001.docx]

**Supplementary methods**

**Study cohorts**

In GEL, participants with ataxia were defined as those who were recruited to the rare diseases programme if their meta data indicated any one of a normalized specific disease of hereditary ataxia, had International Classification of Diseases 10 (ICD-10) code G11 (hereditary ataxia) or a Human Phenotype Ontology (HPO) term [^1^](https://paperpile.com/c/AOyoA1/HbcxY) for ataxia (see Table S1). Similarly, participants with neuropathy were defined as those who were recruited to the rare diseases programme with a normalized specific disease of Charcot-Marie-Tooth disease, the most common form of heritable neuropathy, had ICD-10 code G60 (hereditary and idiopathic neuropathy) or a HPO term for peripheral neuropathy (see Table S1). In the UKB, participants with ataxia were identified as those having an ICD-10 code with prefix G11 (hereditary ataxia) while participants with neuropathy were identified based on having the ICD-10 code with prefix G60 after mapping of ICD-10, ICD-9, readv3, and readv2 codings from the HESIN_DIAG, gp_clinical, and death record tables to ICD-10.

Two separate control cohorts were prepared for the symptom and the polygenic score (PGS) focused analyses. In GEL, controls for the symptom analysis were selected from participants in the rare diseases programme who were not identified to have ataxia or neuropathy; whereas controls for the polygenic score analysis additionally excluded participants with any neurology and neurodevelopmental disorders. In the UKB, controls for the symptom analysis were selected from participants without ataxia or neuropathy defined above; whilst controls for the polygenic score analysis excluded participants with any diseases of the nervous system (G00-G99) or any symptoms and signs involving the nervous and musculoskeletal systems (R25-R29). The removal of these neurology participants from controls for the polygenic score analysis was to avoid or minimise bias by the inclusion of participants whose disease may be associated with undetected pathogenic repeat expansion disease.

We focused on fully consented unrelated participants of European ancestry, as defined by genetic ancestry principal component analysis. In GEL, this was done by excluding participants of non-European ancestry or unknown ancestry (i.e. genetically inferred ancestry posterior probability < 0.8); in UKB, participants of non-European ancestry who were genetically inferred by the Pan-UK Biobank project [^2^](https://paperpile.com/c/AOyoA1/6qyK5) were removed. Participants with unknown and abnormal karyotyped sex (sex chromosome aneuploidy), mismatched sex between self-reported and genetic sex, or without genotypic data were also removed. In GEL, a further exclusion criterion was applied, with participants whose samples were not from blood, did not undergo EDTA extraction, or did not undergo TruSeq PCR-free library preparation, also being excluded.

Related participants up to third degree of relatedness were removed from the study. When individuals with ataxia or neuropathy that have two copies of the pathogenic *RFC1* AAGGG repeat expansions (biallelic affected individuals) were related, one of each pair of the related biallelic affected individuals was randomly excluded. When participants that had biallelic *RFC1* AAGGG repeat expansions, but did not have ataxia or neuropathy (biallelic carriers) were related to biallelic affected individuals, we removed the biallelic carriers; when two biallelic carriers are related, we randomly excluded one of the related pair of the biallelic carriers. This strategy retained as many affected individuals as possible. We excluded controls that were related to biallelic affected individuals or biallelic carriers; when two controls were related, we randomly excluded one of the related pair of controls.

Participants with cerebellar ataxia and/or neuropathy were older than those without cerebellar ataxia or neuropathy in GEL, driven primarily by targeted recruitment of probands with congenital or childhood onset disorders. To remove the potential bias due to the difference in age, we attempted to age-match the controls and biallelic carriers to the age at last clinical investigation (UKB) or current age in the year 2025 (GEL) of the biallelic affected individuals, downsampling to the minimal required amount. We note that the biallelic carriers in GEL could not be completely age-matched due to a difference in the age distribution of this group compared to the biallelic affected individuals. Statistical comparison of age group distribution was performed using an unpaired Wilcoxon test.

**Determination of biallelic *RFC1* AAGGG repeat expansion status**

*RFC1* AAGGG repeat size was genotyped using ExpansionHunter v5.0.0 [^3^](https://paperpile.com/c/AOyoA1/n0kLS) for each participant in GEL and UKB identified above and confirmed with REViewer (full details in the supplementary methods). A subset of individuals in GEL were validated with long-read sequencing data. A kmer-filter was applied to exclude false positive AAGGG calls, as previously described [^4^](https://paperpile.com/c/AOyoA1/mO8oq). Participants with both alleles genotyped as >=30 repeats of the *RFC1* AAGGG motif were short-listed for further analysis. REViewer [^5^](https://paperpile.com/c/AOyoA1/Nt4op) was used to visualize alignments of reads at this locus and plots were visually inspected to confirm if participants had two (biallelic), one or zero copies of the *RFC1* AAGGG RE.

We leveraged long read sequencing data available in GEL to determine the accuracy of our combined ExpansionHunter and REViewer method for determining AAGGG allele status from short read data. The *RFC1* AAGGG allele was characterized using TRGT v4.1.0 [^6^](https://paperpile.com/c/AOyoA1/SdY4d) for the PacBio samples, and longTR v1.2 [^7^](https://paperpile.com/c/AOyoA1/vnPAj) for the ONT samples, using default parameters. Alleles identified were confirmed visually using IGV [^8^](https://paperpile.com/c/AOyoA1/T6GUo).

Biallelic individuals in GEL, UKB and AAC were screened for pathogenic and likely pathogenic variants in genes associated with ataxia or neuropathy (Green List genes in PanelApp Australia, accessed on 17-08-2025) and known REs, to eliminate alternative known monogenic causes for ataxia and neuropathy.

**Symptom analysis**

Participants with cough were defined as those with ICD-10 code R05 (includes Acute cough R05.1, Subacute cough R05.2, Chronic cough R05.3, Cough syncope R05.4, Other specific cough R05.8, and unspecified Cough R05.9) and participants with disorders of vestibular function were defined as those with ICD-10 code H81 (includes Ménière disease H81.0, Benign paroxysmal vertigo H81.1, Vestibular neuronitis H81.2, Other peripheral vertigo H81.3, Vertigo of central origin, Other disorders of vestibular function H81.8, and unspecified Disorder of vestibular function H81.9). Cough or disorders of vestibular function were not collected outside of the context of specific ataxia types in the replication AAC cohort, hence the risk of developing cough or disorders of vestibular function was only assessed in the biallelic affected individuals and biallelic carriers compared to controls in the discovery cohorts.

**Polygenic scores analyses**

Genotypic data of participants in GEL were extracted from the masked aggregated germline variants in the pgen/pvar/psam file format, in which low-quality genotypes were masked by setting them to missing. Specifically for autosomes, genotypes having DP<10 or genotype quality (GQ) <20 or heterozygote genotypes failing an AB ratio binomial test with P-value < 10^-3^ were masked as described by GEL (<https://re-docs.genomicsengland.co.uk/aggv2/>). Genotypic data of UKB participants were genotyped using the SNP array data and imputed using the Haplotype Reference Consortium and UK10K haplotype resource as the reference. Genetic variants for affected individuals and controls in the AAC cohort were jointly called using GATK best practices . rsIDs of variants were added using bcftools [^9^](https://paperpile.com/c/AOyoA1/geoaM).

In all three cohorts genetic variants with a missing rate ≥ 10%, minor allele frequency (MAF) < 0.5%, or deviation from the Hardy-Weinberg equilibrium (HWE, p < 1 × 10^-5^ threshold) were excluded. Precomputed principal components were used for both GEL, using high confidence independent SNPs detailed in the GEL Research Environment User Guide (<https://re-docs.genomicsengland.co.uk/principal_components/>), and the UKB (data field 22009). In AAC, somalier v0.2.19 was used [^10^](https://paperpile.com/c/AOyoA1/rFY8u), to generate the first six ancestry PCs.

Twenty independent lead genetic variants achieving genome-wide significant association with age at total functional capacity score of 6 (TFC6) of Huntington’s disease, after adjusting for the length of the causal CAG *HTT* repeat expansion, were extracted from the latest Genetic Modifiers of Huntington’s Disease Consortium study (GeM-HD 2025) [^11^](https://paperpile.com/c/AOyoA1/KVcgG). According to best practice, rare variants with MAF less than 1-5% are typically excluded from PGS analysis for studies with a sample size of 100 individuals. However, our smallest group is only 18 individuals, and we therefore opted for a more stringent MAF threshold in order to avoid variants with a MAF=0 (monomorphic), which would violate Hardy-Weinberg equilibrium (HWE). Only variants with a MAF ≥ 10% in the GeM-HD 2025 study were included in the calculation of polygenic scores. For lead variants that were missing or did not pass QCs in the discovery and replication cohorts, a proxy variant was chosen as replacement such that it is in high linkage disequilibrium (LD; r^2^ ≥ 0.8) with the unusable lead variant and was genome-wide significantly associated with TFC6 in GeM-HD 2025 study and included in polygenic score calculation. A final set of nine independent lead/proxy variants were included to create polygenic scores. Variant weights were inverted from the age at TFC6 GeM-HD 2025 GWAS betas, such that positive weights corresponded to decreasing functional capacity score, or more severe or faster progressing HD.

QCs of variants were performed using 2.0 ([www.cog-genomics.org/plink/2.0/](http://www.cog-genomics.org/plink/2.0/)) [^12^](https://paperpile.com/c/AOyoA1/hKXTl) and calculation of polygenic scores using Plink 1.9 (<http://pngu.mgh.harvard.edu/purcell/plink/>) [^13^](https://paperpile.com/c/AOyoA1/fwYbQ).

Participants with neurological diseases were excluded from controls for polygenic score (PGS) analyses to avoid or minimise bias by the inclusion of participants whose disease may be associated with undetected pathogenic repeat expansion disease. PGS of biallelic affected individuals and carriers were normalized to have a mean of 0 and standard deviation of 1 based on the age-matched (where possible) controls’ PGS distribution. Associations of disease risk between normalized PGS of biallelic affected individuals and controls, biallelic carriers and controls, were tested using Firth’s logistic regression[^14^](https://paperpile.com/c/AOyoA1/Zz9xC) implemented in the logistf R package v1.26.1, with adjustment for the first 6 ancestry principal components (PCs). We also split participants into quartiles based on the z-score of the standard normal distribution in the assessment of odds ratio (OR) of disease risk of the other quartiles compared to the bottom quartile (the quartile with the lowest PGS as the reference) between biallelic affected individuals and controls, and between biallelic carriers and controls.

Inflammatory bowel disease (IBD) and asthma are not comorbid with *RFC1*-related disease and have PGS available from well-powered GWAS [^15,16^](https://paperpile.com/c/AOyoA1/CJ6C8+PEcIW). Polygenic risk scores for both IBD and asthma were also generated and assessed for their associations with *RFC1*-related disease as negative controls in both the discovery and replication cohorts.

We estimated the associations of disease risk between normalized PGS of biallelic affected individuals and controls, biallelic carriers and controls, and ORs in the two discovery cohorts separately, and then combined these results using a fixed-effect model.

**Single variant association analyses**

We conducted single variant association analyses between disease risk and genotypes of individual SNPs by combining the genotype counts in GEL and UKB using the χ² test for independence, using a permutation test when asymptotic properties were no longer applicable, without ancestry correction. P-values of the χ² test were adjusted using the Benjamini-Hochberg method to correct for multiple testing. For significant associations detected in the discovery cohorts, we attempted to replicate these in the AAC cohort. For each variant, we also calculated odds ratios (ORs) of disease risk for participants that are heterozygous or homozygous for the effect allele compared to those that are homozygous for the reference allele.

**Analysis of age at onset/diagnosis**

We aimed to investigate the link between the TFC6 HD polygenic score and age at onset of the *RFC1*-related disease. However, data of age at onset is not available in the two discovery cohorts. Therefore, we used age at diagnosis of the corresponding normalized specific disease recruited to GEL; whereas in UKB, we used the field G11 first reported (hereditary ataxia; data field 131014) and Date G60 first reported (hereditary and idiopathic neuropathy; 131082) to calculate the age at first report of G11 or G60, and used age at first report for the analysis. In the AAC cohort we used age at onset of symptoms other than cough for the analysis. Since the AAC cohort is an affected-only cohort, raw instead of normalized polygenic scores were analyzed. We conducted the analysis using robust linear models implemented in the robustbase v0.99.4.1 R package.

The first six PCs were adjusted for in the model. There may be a difference in the age at onset/diagnosis for ataxia and neuropathy; therefore, we additionally included disease type (ataxia, neuropathy, or both) as a covariate. To increase power, we conducted a final analysis combining association study results from all three cohorts (two discovery and the replication cohort).

Table S1 HPO terms used to identify ataxia and neuropathy in Genomics England (GEL).

| disease | HPO term | disease subtype |
| --- | --- | --- |
| ataxia | HP:0001251 | Ataxia |
|  | HP:0001310 | Dysmetria |
|  | HP:0002406 | Limb dysmetria |
|  | HP:0020035 | Lower limb dysmetria |
|  | HP:0020036 | Upper limb dysmetria |
|  | HP:0010867 | Dyssynergia |
|  | HP:0010869 | Asynergia |
|  | HP:0010868 | Ocular dyssynergia |
|  | HP:0002066 | Gait ataxia |
|  | HP:0012651 | Abasia |
|  | HP:0007240 | Progressive gait ataxia |
|  | HP:0002078 | Truncal ataxia |
|  | HP:0007221 | Progressive truncal ataxia |
|  | HP:0009878 | Cerebellar ataxia associated with quadrupedal gait |
|  | HP:0002075 | Dysdiadochokinesis |
|  | HP:0002131 | Episodic ataxia |
|  | HP:0002070 | Limb ataxia |
|  | HP:0002470 | Nonprogressive cerebellar ataxia |
|  | HP:0031868 | Optic ataxia |
|  | HP:0002073 | Progressive cerebellar ataxia |
|  | HP:0002497 | Spastic ataxia |
| neuropathy | HP:0009830 | Peripheral neuropathy |
|  | HP:0000763 | Sensory neuropathy |
|  | HP:0007067 | Distal peripheral sensory neuropathy |
|  | HP:0003409 | Distal sensory impairment of all modalities |
|  | HP:0003434 | Sensory ataxic neuropathy |
|  | HP:0003390 | Sensory axonal neuropathy |
|  | HP:0007108 | Demyelinating peripheral neuropathy |
|  | HP:0007131 | Acute demyelinating polyneuropathy |
|  | HP:0007220 | Demyelinating motor neuropathy |
|  | HP:0011402 | Demyelinating sensory neuropathy |
|  | HP:0012181 | Entrapment neuropathy |
|  | HP:0012185 | Constrictive median neuropathy |
|  | HP:0012216 | Entrapment neuropathy of suprascapular nerve |
|  | HP:0012186 | Entrapment neuropathy of the ulnar nerve at elbow |
|  | HP:0003477 | Peripheral axonal neuropathy |
|  | HP:0007267 | Chronic axonal neuropathy |
|  | HP:0007002 | Motor axonal neuropathy |
|  | HP:0003390 | Sensory axonal neuropathy |
|  | HP:0007178 | Motor polyneuropathy |
|  | HP:0007220 | Demyelinating motor neuropathy |
|  | HP:0003489 | Acute episodes of neuropathic symptoms |
|  | HP:0001301 | Chronic sensorineural polyneuropathy |
|  | HP:0006903 | Congenital peripheral neuropathy |
|  | HP:0006949 | Episodic peripheral neuropathy |
|  | HP:0007327 | Mixed demyelinating and axonal polyneuropathy |
|  | HP:0009831 | Mononeuropathy |
|  | HP:0032018 | Multiple mononeuropathy |
|  | HP:0001271 | Polyneuropathy |
|  | HP:0007133 | Progressive peripheral neuropathy |
|  | HP:0007141 | Sensorimotor neuropathy |
|  | HP:0006865 | Sensorimotor polyneuropathy affecting arms more than legs |

Table S2 Number and percentage of visually inspected biallelic *RFC1* AAGGG participants by ranges of the short allele of ExpansionHunter genotyped *RFC1* AAGGG repeat motif in GEL and UKB.

| Cohort | Short allele of *RFC1*:AAGGG genotypes by EH5 | Total number of participants | Number of biallelic *RFC1*:AAGGG participants | Percentage of biallelic participants |
| --- | --- | --- | --- | --- |
| UKB | >= 30 but < 40 | 319 | 14 | 4.4% |
|  | >= 40 but < 45 | 515 | 26 | 5.0% |
|  | >= 45 but < 50 | 484 | 52 | 10.7% |
|  | >= 50 but < 55 | 257 | 63 | 24.5% |
|  | >= 55 | 187 | 80 | 42.8% |
| GEL | >= 30 but < 40 | 81 | 1 | 1.2% |
|  | >= 40 but < 45 | 93 | 6 | 6.5% |
|  | >= 45 but < 50 | 109 | 9 | 8.3% |
|  | >= 50 but < 55 | 95 | 19 | 20.0% |
|  | >= 55 | 158 | 99 | 62.7% |

Table S3. Lead SNPs from GeM-HD and their inclusion/exclusion status.

| SNV | Candidate gene | EA | GeM-HD 2025 | | | inclusion/exclusion |
| --- | --- | --- | --- | --- | --- | --- |
|  |  |  | MAF | beta | pval |  |
| rs6434365 | *PMS1* | C | 0.209 | -0.12 | 5.85E-14 | Included |
| rs148648107 | *MLH1* | A | 0.305 | 0.14 | 8.01E-23 | Excluded, no proxy SNP identified* |
| rs245100 | *MSH3* | G | 0.249 | -0.23 | 5.63E-57 | Included |
| rs6151716 | *MSH3* | G | 0.16 | 0.22 | 3.36E-36 | Proxy SNP used:  rs1650735 |
| rs113361582 | *MSH3* | G | 0.003 | 0.9 | 9.14E-18 | Excluded (MAF<10%)** |
| rs202157262 | *TCERG1* | A | 0.02 | 0.25 | 3.29E-08 | Excluded (MAF<10%) |
| rs79727797 | *TCERG1* | A | 0.027 | 0.32 | 1.83E-18 | Excluded (MAF<10%) |
| rs1805326 | *PMS2* | T | 0.148 | 0.15 | 1.06E-16 | Proxy SNP used:  rs12531376 |
| rs1805324 | *PMS2* | T | 0.021 | -0.26 | 3.47E-09 | Excluded (MAF<10%) |
| rs2345058 | *PMS2* | C | 0.409 | 0.12 | 1.82E-19 | Included |
| rs79136984 | *RRM2B* | T | 0.078 | -0.16 | 7.88E-12 | Excluded (MAF<10%) |
| rs12365516 | *CCDC82* | T | 0.196 | 0.11 | 9.99E-13 | Included |
| rs151322829 | *FAN1* | T | 0.008 | -0.64 | 3.02E-21 | Excluded (MAF<10%) |
| rs150393409 | *FAN1* | A | 0.012 | -0.87 | 2.69E-52 | Excluded (MAF<10%) |
| rs118089305 | *FAN1* | T | 0.018 | -0.31 | 8.63E-12 | Excluded (MAF<10%) |
| rs3512 | *FAN1* | C | 0.311 | 0.22 | 1.08E-53 | Included |
| rs145821638 | *LIG1* | A | 0.002 | 1.24 | 4.46E-16 | Excluded (MAF<10%) |
| rs3078205 | *LIG1* | A | 0.13 | 0.15 | 1.59E-15 | Proxy SNP used:  rs10411910 |
| rs112837068 | *POLD1* | C | 0.097 | 0.13 | 6.62E-10 | Excluded (MAF<10%) |
| rs165666 | *MED15* | A | 0.213 | -0.09 | 5.03E-09 | Included |

*Some SNPs are missing from one or more of the cohorts, due to issues such as quality filtering. To harmonise SNPs between the three cohorts, proxy SNPs in high LD with the lead SNPs and present in all three cohorts were used in place of the lead variant, were relevant. No suitable proxy SNPs were identified for SNP rs148648107, and it was excluded from the analysis. ** Variants with a MAF<10% in GeM-HD were excluded from our PGS analysis, as this study is too underpowered to reliably detect rare or less common variants.

Table S4 Normalised specific disease of 13 of the 26 biallelic carriers who were recruited to the rare diseases programme as probands in GEL.

| Normalised specific disease | count |
| --- | --- |
| Congenital muscular dystrophy | 1 |
| Cystic kidney disease | 1 |
| Early onset and familial Parkinson's Disease | 1 |
| Familial Hypercholesterolaemia | 1 |
| Familial Thoracic Aortic Aneurysm Disease | 1 |
| Insulin resistance (including lipodystrophy) | 1 |
| Intellectual disability | 1 |
| Long QT syndrome | 1 |
| Mitochondrial disorders | 1 |
| Neurofibromatosis Type 1 | 1 |
| Primary lymphoedema | 1 |
| Rhabdomyolysis and metabolic muscle disorders | 1 |
| Rod-cone dystrophy | 1 |

Figure S1 Number of biallelic affected individuals identified by disease types in 3 cohorts. Total number of individuals with ataxia and/or neuropathy in GEL (a) and UKB (b); number of unrelated biallelic affected individuals with European ancestry in GEL (c), UKB (d), and AAC (e), with ataxia, neuropathy, or both. Values in the parentheses are the percentage of unrelated biallelic affected individuals in the total number of individuals by disease, e.g. 25 biallelic affected individuals with neuropathy and ataxia account for 10.5% of 238 individuals with both diseases in GEL.

Figure S2. Age matching in UKB and GEL in the final cohort for the PGS analysis. Comparison of the current age, age at last investigation and age at ataxia or neuropathy last diagnosis (where relevant) in the UKB is shown for biallelic affected individuals (a), biallelic carriers (b) and controls (c). Plot (d) shows the comparison of the age at last investigation in UKB of biallelic carriers and controls compared to the age at ataxia or neuropathy diagnosis. In GEL, the boxplot shows the comparison of current age in biallelic affected individuals, biallelic carriers and controls, as well as the age at ataxia or neuropathy diagnosis for the biallelic affected individuals. All current ages are calculated for the year 2025. P-values are from unpaired Wilcoxon tests. Outliers not plotted.

a

b

Figure S3 Comparison of the detection of *RFC1*:AAGGG alleles in short- and long-read sequencing WGS data in individuals from GEL. Plots in (a) show the allele size estimates for *RFC1*:AAGGG alleles detected in ONT (top) and PacBio (bottom) WGS sequencing data. Blue symbols indicate individuals called as biallelic in by EH5 and REViewer in the short read data, while red indicates individuals with a single allele. Triangles indicate individuals identified as biallelic in ONT or PacBio, while circles indicate those that have a single AAGGG allele. The data are divided by allele status in affected individuals and controls as determined by short-read sequencing, and other indicates individuals who are in the rare disease group in GEL but were not included in the final cohort in this study (due to factors such as relatedness, ethnicity, age etc as detailed in the methods section). Plots in (b) show a comparison of allele size for individuals sequenced on both the ONT and PacBio platforms, with red indicating the *RFC1* AAGGG allele status determined by EH5 and REViewer.

Figure S4 Differences in the mean PGS amongst biallelic affected individuals, carriers, and controls in the discovery cohorts (a, b) and *RFC1* related disease risk of biallelic carriers compared to controls estimated by the Firth’s logistic regression (c).

 Figure S5 Forest plot of odds ratio estimated by Firth’s logistic regression using PGS of IBD/asthma in the discovery and validation cohort in biallelic affected individuals (top) and carriers (bottom).

**References**

1.	[Gargano MA, Matentzoglu N, Coleman B, et al. The Human Phenotype Ontology in 2024: phenotypes around the world. Nucleic Acids Res. 2024;52(D1):D1333–D1346.[cited 2025 Sep 9 ]](http://paperpile.com/b/AOyoA1/HbcxY)

2.	[Karczewski KJ, Gupta R, Kanai M, et al. Pan-UK Biobank GWAS improves discovery, analysis of genetic architecture, and resolution into ancestry-enriched effects [Internet]. bioRxiv 2024;2024.03.13.24303864.[cited 2025 Sep 9 ] Available from:](http://paperpile.com/b/AOyoA1/6qyK5) <https://www.medrxiv.org/content/10.1101/2024.03.13.24303864v2.abstract>

3.	[Dolzhenko E, Deshpande V, Schlesinger F, et al. ExpansionHunter: a sequence-graph-based tool to analyze variation in short tandem repeat regions. Bioinformatics 2019;35(22):4754–4756.[cited 2025 Sep 9 ]](http://paperpile.com/b/AOyoA1/n0kLS)

4.	[Rafehi H, Fearnley LG, Read J, et al. A prospective trial comparing programmable targeted long-read sequencing and short-read genome sequencing for genetic diagnosis of cerebellar ataxia. Genome Res. 2025;35(4):769–785.[cited 2025 Aug 13 ]](http://paperpile.com/b/AOyoA1/mO8oq)

5.	[Dolzhenko E, Weisburd B, Ibañez K, et al. REViewer: haplotype-resolved visualization of read alignments in and around tandem repeats. Genome Med. 2022;14(1):84.[cited 2025 Sep 9 ]](http://paperpile.com/b/AOyoA1/Nt4op)

6.	[Dolzhenko E, English A, Dashnow H, et al. Characterization and visualization of tandem repeats at genome scale. Nat. Biotechnol. 2024;42(10):1606–1614.[cited 2024 Nov 26 ]](http://paperpile.com/b/AOyoA1/SdY4d)

7.	[Ziaei Jam H, Zook JM, Javadzadeh S, et al. LongTR: genome-wide profiling of genetic variation at tandem repeats from long reads. Genome Biol. 2024;25(1):176.[cited 2025 Nov 15 ]](http://paperpile.com/b/AOyoA1/vnPAj)

8.	[Robinson JT, Thorvaldsdóttir H, Winckler W, et al. Integrative genomics viewer. Nat. Biotechnol. 2011;29(1):24–26.[cited 2025 Oct 20 ]](http://paperpile.com/b/AOyoA1/T6GUo)

9.	[Danecek P, Bonfield JK, Liddle J, et al. Twelve years of SAMtools and BCFtools [Internet]. Gigascience 2021;10(2)[cited 2025 Sep 9 ] Available from:](http://paperpile.com/b/AOyoA1/geoaM) <http://dx.doi.org/10.1093/gigascience/giab008>

10.	[Pedersen BS, Bhetariya PJ, Brown J, et al. Somalier: rapid relatedness estimation for cancer and germline studies using efficient genome sketches. Genome Med. 2020;12(1):62.[cited 2025 Sep 9 ]](http://paperpile.com/b/AOyoA1/rFY8u)

11.	[Genetic Modifiers of Huntington’s Disease (GeM-HD) Consortium, Group 1, Lee J-M, et al. Genetic modifiers of somatic expansion and clinical phenotypes in Huntington’s disease highlight shared and tissue-specific effects [Internet]. Nat. Genet. 2025;[cited 2025 Jun 11 ] Available from:](http://paperpile.com/b/AOyoA1/KVcgG) <http://dx.doi.org/10.1038/s41588-025-02191-5>

12.	[Chang CC, Chow CC, Tellier LC, et al. Second-generation PLINK: rising to the challenge of larger and richer datasets. Gigascience 2015;4(1):7.[cited 2025 Sep 9 ]](http://paperpile.com/b/AOyoA1/hKXTl)

13.	[Purcell S, Neale B, Todd-Brown K, et al. PLINK: a tool set for whole-genome association and population-based linkage analyses. Am. J. Hum. Genet. 2007;81(3):559–575.[cited 2025 Sep 9 ]](http://paperpile.com/b/AOyoA1/fwYbQ)

14.	[Heinze G, Schemper M. A solution to the problem of separation in logistic regression. Stat. Med. 2002;21(16):2409–2419.](http://paperpile.com/b/AOyoA1/Zz9xC)

15.	[Demenais F, Margaritte-Jeannin P, Barnes KC, et al. Multiancestry association study identifies new asthma risk loci that colocalize with immune-cell enhancer marks. Nat. Genet. 2018;50(1):42–53.[cited 2025 Jul 28 ]](http://paperpile.com/b/AOyoA1/CJ6C8)

16.	[de Lange KM, Moutsianas L, Lee JC, et al. Genome-wide association study implicates immune activation of multiple integrin genes in inflammatory bowel disease. Nat. Genet. 2017;49(2):256–261.[cited 2025 Jul 25 ]](http://paperpile.com/b/AOyoA1/PEcIW)
